# An 8‐Week Triple‐Blind, Randomized, Placebo‐Controlled Trial Evaluating High‐Dose Vitamin D3 and Flaxseed Oil for Inflammation and Metabolic Health in Elderly Patients With Type 2 Diabetes: Implications for Clinical Management

**DOI:** 10.1155/jnme/8892400

**Published:** 2026-07-29

**Authors:** Naser Aghamohammadzadeh, Mojgan Abdi Oskoui, Neda Dolatkhah, Azizeh Farshbaf Khalili

**Affiliations:** ^1^ Endocrine Research Center, Tabriz University of Medical Sciences, Tabriz, Iran, tbzmed.ac.ir; ^2^ Faculty of Medicine, Tabriz University of Medical Sciences, Tabriz, Iran, tbzmed.ac.ir; ^3^ Research Center for Nutritional Sciences, Iran University of Medical Sciences, Tehran, Iran, iums.ac.ir; ^4^ Department of Nutrition, School of Public Health, Iran University of Medical Sciences, Tehran, Iran, iums.ac.ir; ^5^ Physical Medicine and Rehabilitation Research Center, Tabriz University of Medical Sciences, Tabriz, Iran, tbzmed.ac.ir

**Keywords:** flaxseed oil, inflammation, oxidative stress, Type 2 diabetes mellitus, vitamin D3

## Abstract

**Aims:**

This study aimed to evaluate the effects of cosupplementation with high‐dose vitamin D3 and flaxseed oil on inflammatory markers, oxidative stress, and metabolic health in elderly patients with T2DM.

**Materials and Methods:**

We conducted an 8‐week, triple‐blind, randomized, placebo‐controlled clinical trial in Iran, enrolling 104 elderly patients with T2DM, of whom 102 completed the study. The intervention group received 500 mg of flaxseed oil three times daily and 50,000 IU of vitamin D3 orally every two weeks, while the control group received matching placebos.

**Results:**

The primary outcome, serum hs‐CRP, was significantly reduced in the intervention group compared to controls (MD: −0.98 μg/mL, *p* = 0.034). Exploratory analyses of secondary outcomes suggested additional potential benefits: total antioxidant capacity improved (MD: +0.60 mmol/L, *p* < 0.05), and reductions were observed in weight (MD: −1.73 kg, *p* < 0.001), BMI (MD: −0.67 kg/m^2^, *p* < 0.001), fasting blood glucose (MD: −22.83 mg/dL, *p* = 0.015), fasting insulin (MD: −0.94 μIU/mL, *p* = 0.008), homeostatic model assessment of insulin resistance (MD: −1.31, *p* = 0.001), triglycerides (MD: −15.00 mg/dL, *p* = 0.020), and total cholesterol (MD: −2.52 mg/dL, *p* = 0.028). Physical health‐related quality of life, including pain scores and physical functioning, also improved significantly (*p* < 0.05). The intervention was well tolerated with minimal adverse events.

**Conclusion:**

Cosupplementation with flaxseed oil and high‐dose vitamin D_3_ significantly reduced systemic inflammation in elderly patients with T2DM. Exploratory findings further suggest potential benefits for oxidative stress, glycemic control, lipid profile, and physical quality of life, warranting confirmation in future trials.

**Trial Registration:** Iranian Clinical Trials Register: IRCT20161022030424N8

## 1. Introduction

Type 2 diabetes mellitus (T2DM) poses significant clinical challenges for the elderly population, primarily due to age‐related physiological changes, multiple comorbidities, polypharmacy, and malnutrition risk. As prevalence increases markedly with age, individualized care strategies are essential. Given the rapidly growing aging population, effective management of T2DM in older adults remains a critical public health priority [1, 2].

Aging in patients with T2DM is closely associated with elevated oxidative stress and chronic low‐grade inflammation, both of which worsen progressively with age, contributing to accelerated functional decline and increased risk of complications [3]. Notably, cyclooxygenase (COX)‐mediated inflammatory pathways, characterized by increased prostaglandin metabolites, further amplify this burden [4]. While lifestyle interventions remain essential, certain antidiabetic agents—including pioglitazone, metformin, and glucagon‐like peptide‐1 receptor (GLP‐1R) agonists—exhibit ancillary anti‐inflammatory properties. Nevertheless, the efficacy of pharmacological and nutritional interventions in reducing oxidative stress and inflammation in elderly patients with T2DM remains inconclusive, highlighting a critical gap in current diabetes management.

Nutritional approaches play a vital role in modulating inflammatory responses among older adults with T2DM. Recent guidelines emphasize a shift from merely preventing metabolic syndrome toward addressing frailty, with particular focus on adequate energy intake, sufficient protein and micronutrient consumption, and adherence to anti‐inflammatory dietary patterns [5]. Among relevant micronutrients, vitamin D merits special attention given its immunomodulatory properties and the high prevalence of deficiency in older adults, particularly those with T2DM and polypharmacy. Disruptions in hepatic or renal hydroxylation, as well as enzymatic dysregulation, can compromise circulating 25‐hydroxyvitamin D levels and downstream bioactivation to calcitriol. Complementing this, emerging evidence suggests that n‐3 fatty acids—particularly alpha‐linolenic acid (ALA)—may enhance vitamin D bioactivation, potentially amplifying its anti‐inflammatory and immunomodulatory effects [6].

The novelty of this study lies in examining the combined effects of high‐dose vitamin D3 and flaxseed oil—a plant‐based source of ALA—in elderly patients with T2DM. Recent investigations have highlighted the potential benefits of high‐dose vitamin D3 and omega‐3 fatty acid cosupplementation for managing chronic conditions, including cancer. Mechanistically, the combination of n‐3 fatty acids and vitamin D may improve pancreatic β‐cell function [7]. However, marine‐derived omega‐3 sources, such as fish oil, are frequently poorly tolerated due to gastrointestinal discomfort. Consequently, plant‐based alternatives—particularly flaxseed oil, which contains approximately 50% ALA—have gained increasing attention [8], with emerging evidence supporting their role in glycemic control [9–11].

Flaxseed oil supplementation has demonstrated beneficial effects on various health biomarkers. Meta‐analyses have established its antioxidant properties, reporting reductions in malondialdehyde (MDA) levels and improvements in total antioxidant capacity (TAC) [12, 13]. However, findings regarding its effects on glycemic control and inflammation in T2DM remain inconsistent. While some trials report benefits when flaxseed oil is combined with vitamin D in diabetic patients with comorbidities such as coronary heart disease (CHD), others demonstrate limited or no effects on insulin sensitivity or glycemic outcomes [14–17]. Comparable inconsistencies have been observed with vitamin D monotherapy. These discrepancies underscore the need for further investigation, particularly in elderly patients who face unique metabolic challenges. Nevertheless, cosupplementation has shown promising results in specific populations—including those with cancer or metabolic syndrome—with notable improvements in inflammatory markers and body composition indices [14, 18].

While the consequences of poor glycemic control are well established, insulin resistance and chronic inflammation represent fundamental underlying mechanisms warranting greater clinical attention. Despite growing interest in the anti‐inflammatory and antioxidative potential of vitamin D and ALA, few studies have specifically examined their combined effects in elderly patients with T2DM—a population uniquely vulnerable to malnutrition and chronic inflammation. Therefore, this study aimed to evaluate whether cosupplementation with high‐dose vitamin D3 and flaxseed oil exerts synergistic effects on inflammatory markers and oxidative stress in this at‐risk population.

Beyond glycemic control and inflammatory indices, lipid dysregulation represents a central feature of T2DM in elderly patients, closely intertwined with insulin resistance, oxidative stress, and systemic inflammation [19]. Elevated triglycerides, reduced high‐density lipoprotein cholesterol (HDL‐C), and increased low‐density lipoprotein cholesterol (LDL‐C) are commonly observed in this population and contribute to heightened cardiovascular risk [20]. Notably, both vitamin D deficiency and ALA have been independently associated with adverse lipid profiles, and their correction may favorably influence lipid metabolism through anti‐inflammatory and antioxidative pathways [21, 22]. Furthermore, the cumulative burden of chronic inflammation, oxidative stress, and metabolic dysfunction carries profound implications for health‐related quality of life (HRQoL) in this population [23]. Impaired glycemic control and systemic inflammation are established contributors to fatigue, physical limitation, and psychological distress in elderly patients with T2DM [24]. Therefore, alongside biochemical outcomes, this study assessed lipid profiles and HRQoL as secondary outcomes, recognizing that meaningful clinical improvement in this population extends beyond laboratory markers to encompass functional and subjective well‐being.

This investigation addresses a timely and clinically important gap by providing novel insights into nutritional strategies for managing T2DM in a vulnerable population. By examining the interplay between oxidative stress, inflammation, and glycemic control, the findings may inform future clinical practice and contribute to improved health outcomes in elderly patients with T2DM.

## 2. Methods

### 2.1. Study Design and Participants

**FIGURE 1:**
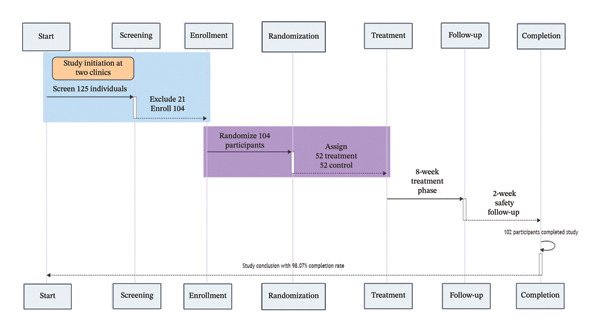
Participant recruitment, screening, and enrollment.

This parallel‐group, triple‐blind randomized clinical trial (RCT) was conducted at two outpatient clinics affiliated with the Tabriz University of Medical Sciences—Imam Reza and Sina hospitals, Iran—between March 2024 and June 2025. The trial evaluated the efficacy and safety of cosupplementation with high‐dose vitamin D3 and flaxseed oil in elderly patients with T2DM. The study comprised a screening period of up to 3 weeks, an 8‐week intervention phase, and a 2‐week safety follow‐up (Figure 1). The protocol was approved by the Ethics Committee of Tabriz University of Medical Sciences (IR.TBZMED.REC.1402.210) in accordance with the Declaration of Helsinki and registered with the Iranian Clinical Trials Register. Written informed consent was obtained from all participants before enrollment.

Informed consent was obtained through a structured process in which participants received a comprehensive information sheet outlining the study objectives, intervention details, expected duration, and potential risks and benefits. Participants were encouraged to ask questions and seek clarification before providing consent.

Eligible participants were men and women aged ≥ 65 years with a confirmed diagnosis of T2DM, defined as HbA1c ≥ 6.5% or fasting blood glucose (FBG) ≥ 126 mg/dL [25]. Additional inclusion criteria required serum 25‐hydroxyvitamin D levels between 30 and 125 nmol/L and a stable antidiabetic regimen for at least 2 months prior to enrollment, with no more than a 10% adjustment in insulin dosage where applicable.

Exclusion criteria included other forms of diabetes, serious cardiovascular or cerebrovascular events within 3 months prior to randomization, and serum 25‐hydroxyvitamin D levels outside the specified range. Participants were also excluded if they had a history of cancer, severe liver dysfunction, or chronic kidney disease, or if they were current smokers, alcohol consumers, systemic steroid users, or had participated in another interventional trial within the preceding 3 months.

The participant recruitment and selection process are summarized in Figure 1.

### 2.2. Sample Size

Sample size was determined based on the primary outcome, serum hs‐CRP, using data from Raygan et al., [26] who reported a significant reduction in hs‐CRP following flaxseed oil supplementation in patients with T2DM and CHD. From the reported means and standard deviations, a Cohen’s d effect size of 0.55 was calculated, indicating a medium‐to‐large effect. Sample size was then estimated for a two‐tailed independent *t*‐test using the following formula:

(1) n=2Zα/2+Zβ2·σ2Δ2=2Zα/2+Zβ2d2,

where *Z*_*α*/2_ corresponds to the significance level (*α* = 0.05, *Z*_0.05/2_ = 1.96) and *Z*_*β*_ (*Z*_0.20_ = 0.84, 1 − *β* = 0.80) corresponds to the statistical power. The ratio of the expected difference (Δ) to the pooled standard deviation (*σ*) constitutes the effect size (*d* = Δ/*σ* = 0.55).

Using G∗Power software (Version 3.1.9.7), with a Type I error rate (*α* = 0.05) and statistical power (1 − *β* = 0.80), the minimum required sample size was 50 participants per group. Accounting for an anticipated dropout rate of approximately 5%, the target was adjusted to 52 participants per group (104 total). Ultimately, 102 participants completed the study—52 in the intervention group and 50 in the placebo group—meeting the prespecified minimum threshold and ensuring adequate statistical power to detect meaningful changes in hs‐CRP levels.

### 2.3. Randomization, Treatment Allocation, and Blinding

Participants were randomly assigned in a 1:1 ratio to the intervention or placebo group for 8 weeks. The allocation sequence was generated by an independent statistician using Random Allocation Software (RAS) Version 1.0, with no involvement in recruitment, data collection, or outcome assessment. Block randomization with block sizes of 4 and 6 was employed to prevent allocation predictability. Randomization was stratified by two factors: age group (65–74 years and ≥ 75 years), based on the WHO classification distinguishing young–old from old–old adults given their differences in metabolic function and treatment response [27], and baseline FBG level (100–199, 200–299, and ≥ 300 mg/dL), reflecting clinically recognized categories of acceptable, poor, and very poor glycemic control, respectively [28]. FBG stratification was considered essential given that baseline glycemic status may independently influence inflammatory markers, including the primary outcome, hs‐CRP.

Enrollment and group assignment were conducted by a designated study coordinator independent of the randomization process. Allocation concealment was maintained using sealed, consecutively numbered opaque bottles, preventing selection bias and ensuring that group assignments remained unknown to both participants and clinicians until the point of intervention.

Placebos were manufactured by the same companies as the active supplements—Barij Essence for flaxseed oil and Zahravi for vitamin D_3_. The flaxseed oil placebo consisted of 500 mg of liquid paraffin, matched to the active capsule in appearance, color, size, smell, and taste. The vitamin D_3_ placebo contained an inert oil–based substance, identical to the active capsule in shape, size, color, and packaging. Both placebos were dispensed in identically appearing, consecutively numbered opaque bottles.

Participants, clinicians, and outcome assessors remained blinded to group allocations throughout the study, minimizing the risk of performance and detection bias.

### 2.4. Study Intervention and Dosage Justification

Intervention doses were selected based on evidence from prior clinical studies. Flaxseed oil was administered at 1500 mg/day (500 mg three times daily), a dose demonstrated to provide sufficient ALA to modulate inflammatory markers without significant adverse effects [29]. Vitamin D_3_ was administered at 50,000 IU every 2 weeks, following established supplementation protocols aimed at raising serum 25‐hydroxyvitamin D levels into the sufficiency range in deficient populations [30].

Flaxseed oil soft gels were manufactured by Barij Essence Pharmaceutical Co. (Kashan, Iran), with each capsule containing 500 mg of cold‐pressed flaxseed oil standardized to 70% n‐3 polyunsaturated fatty acids (PUFAs) (350 mg ALA).

Vitamin D_3_ supplements (50,000 IU cholecalciferol per pearl) were produced by Zahravi Pharmaceutical Company (Tabriz, Iran).

Matching placebos for both supplements were manufactured by the same respective companies: the flaxseed oil placebo consisted of 500 mg of liquid paraffin, identical to the active capsule in appearance, color, size, smell, and taste, while the vitamin D_3_ placebo contained an inert oil–based substance matched in shape, size, color, and packaging. Both placebos were dispensed in consecutively numbered opaque bottles.

Participants, clinicians, and outcome assessors remained blinded to group allocations throughout the study, minimizing performance and detection bias.

Participants were instructed to maintain their usual medication regimen, physical activity, and dietary habits throughout the intervention period, with any medication changes permitted only when clinically necessary. Supplement adherence was monitored through biweekly pill counts and daily text message reminders. Compliance was defined as the consumption of more than 90% of the assigned supplements.

### 2.5. Outcomes and Other Variables of Interest

#### 2.5.1. Primary Outcome

The primary outcome was the change in the serum hs‐CRP concentration from baseline to week 8, which served as the basis for sample size calculation and the confirmatory hypothesis test.

#### 2.5.2. Secondary Outcomes

Secondary outcomes included changes in additional inflammatory markers (interleukin‐6 [IL‐6] and tumor necrosis factor‐alpha [TNF‐α]), oxidative stress indices (TAC and MDA), anthropometric measurements, lipid profile (total cholesterol, LDL‐C, HDL‐C, and triglycerides), health‐related quality of life, and adverse events. These outcomes were exploratory in nature and are reported descriptively to generate hypotheses for future research; given the multiplicity of comparisons, findings should be interpreted with appropriate caution.

#### 2.5.3. Dietary Intake and Physical Activity Assessment

Dietary intake was assessed using 3‐day 24‐h food recalls (two weekdays and one weekend day) conducted at baseline and week 8 via face‐to‐face or telephone interviews. Data were analyzed by a qualified nutritionist using Nutritionist IV software (First DataBank, San Bruno, CA, USA), adapted for Iranian foods. Physical activity was evaluated at baseline and week 8 using the International Physical Activity Questionnaire (IPAQ), expressed in metabolic equivalents per hour per day (MET‐h/d) [31, 32]. Activity levels were categorized per standard IPAQ scoring criteria: light activity (< 600 MET‐min/week) and moderate activity (600–3000 MET‐min/week).

#### 2.5.4. Measurement of Anthropometric Parameters

Anthropometric measurements were obtained with participants minimally clothed and without shoes. Weight was recorded to the nearest 0.5 kg using a Seca scale (Seca, Hamburg, Germany) and height to the nearest 0.1 cm using a Seca stadiometer. BMI was calculated as weight (kg) divided by height squared (m^2^) and categorized according to WHO standards [33].

#### 2.5.5. Measurement of Biochemical Parameters

Following a 10–12‐h overnight fast, venous blood samples were collected into plain tubes and allowed to clot at room temperature for 10–15 min. Samples were centrifuged at 10,000 rpm for 20 min (Sigma, UK), and the separated serum was stored at −70°C until analysis. Measured parameters included FBG, HbA1c, insulin, hs‐CRP, TAC, and MDA.

Hs‐CRP levels were quantified using the immunoturbidimetric technique with a Pars Azmoon kit (Pars Azmoon Inc., Tehran, Iran) on a Hitachi 917 analyzer, with a sensitivity of 0.1 μg/mL. Serum TAC was assessed using a colorimetric assay kit (Naxifer, Cat# NS‐15012; Navand Salamat Company, Iran). MDA was measured using the Nalondi Lipid Peroxidation Assay Kit (Navand Salamat Company, Iran). FBG was evaluated using the glucose oxidase method on an autoanalyzer (Cobas c 311, Roche Diagnostics, Risch‐Rotkreuz, Switzerland). Serum insulin levels were measured using an enzyme‐linked immunosorbent assay (ELISA) kit (Monobind Inc., Lake Forest, California, USA), while HbA1c levels were determined via high‐performance liquid chromatography (Tosoh, Tokyo, Japan). Insulin resistance was calculated using the homeostatic model assessment for insulin resistance (HOMA‐IR) formula: HOMA‐IR = fasting insulin (μU/mL) × FPG (mmol/L)/22.5. Lipid profiles—including triglycerides, total cholesterol, and HDL‐C—were assessed using enzymatic colorimetric methods with Pars Azmoon kits and an autoanalyzer (Selectra ProXL, Vital Scientific, Spankeren, The Netherlands). LDL‐C concentrations were calculated using the Friedewald equation: LDL‐C (mg/dL) = Total Cholesterol − HDL‐C − (Triglycerides/5) [34]. This equation was applied exclusively to samples with fasting triglyceride levels below 400 mg/dL, a threshold above which the Friedewald equation is considered unreliable. All participants in both groups had triglyceride levels below this threshold at both baseline and postintervention assessments.

#### 2.5.6. Quality of Life Assessment

To evaluate the participants′ physical and mental well‐being, the validated Persian version of the Short‐Form Health Survey (SF‐36) was administered at baseline and after 8 weeks [35]. The SF‐36 is a widely recognized instrument comprising 36 items that assess eight health domains: physical functioning, role‐physical, bodily pain, general health, vitality, social functioning, role‐emotional, and mental health [36]. These domains are further categorized into two summary components: the physical component summary (PCS) and the mental component summary (MCS). Each domain is scored on a scale of 0–100, where higher scores indicate better health status and overall quality of life.

### 2.6. Adverse Events

Adverse events were closely monitored throughout the study, with particular emphasis on gastrointestinal and allergic reactions. All adverse events were systematically documented, including their nature, severity, and duration. This comprehensive monitoring enabled the timely identification and management of any issues arising during the trial. Adverse event reports were compiled and analyzed to assess the safety profile of the intervention, ensuring that potential risks were promptly identified and effectively communicated. Regular safety assessments were conducted to evaluate the overall well‐being of participants.

### 2.7. Statistical Analysis

Statistical analyses were conducted using SPSS Version 22.0 (IBM Corp., Armonk, NY, USA). The normality of quantitative variables was evaluated using both the Kolmogorov–Smirnov test and skewness/kurtosis values. Data were analyzed according to participants′ randomization assignment using an intention‐to‐treat (ITT) approach.

To compare baseline characteristics and biochemical measurements between groups, chi‐squared tests were used for categorical variables, and independent‐sample *t*‐tests (or Mann–Whitney *U* tests for nonnormal data) were used for continuous variables. For evaluating within‐group changes from baseline to postintervention, paired sample *t*‐tests or Wilcoxon signed‐rank tests were employed based on data distribution.

For the primary analysis, a linear mixed model (LMM) was employed, incorporating treatment groups and baseline values as fixed effects and subjects as random effects. Missing data were handled directly within the LMM framework using the restricted maximum‐likelihood (REML) approach under the missing at random (MAR) assumption.

Given the inclusion of 12 secondary outcomes, the Benjamini–Hochberg procedure was applied to control the false discovery rate (FDR) at 5% for all secondary hypotheses, thereby minimizing the risk of Type I error while preserving adequate statistical power. Effect sizes (such as Cohen’s d) were calculated for all key outcomes to evaluate the clinical relevance of the observed differences.

## 3. Results

### 3.1. Baseline Characteristics

**FIGURE 2:**
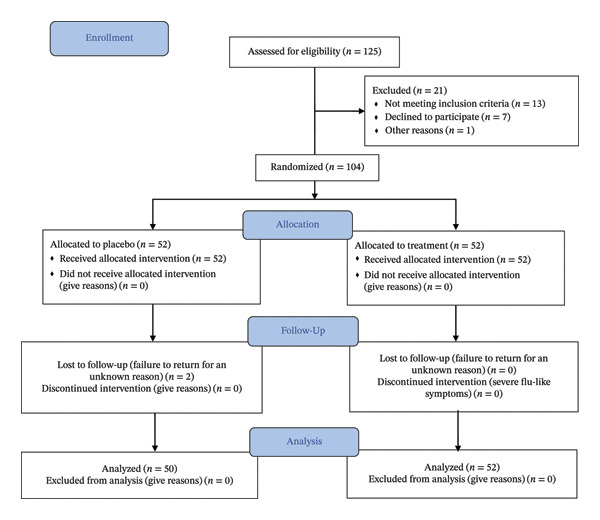
CONSORT 2010 flow diagram.

A total of 125 individuals were screened for eligibility, of whom 104 were enrolled in the study. Twenty‐one individuals were excluded due to not meeting the eligibility criteria, declining participation, or withdrawing consent. The final sample of 104 participants was randomized into a treatment group (*n* = 52) and a control group (*n* = 52). Two participants in the placebo group did not complete the intervention, resulting in 102 participants (98.08%) completing the study (Figure 2).

Baseline characteristics, including age, sex, education, disease duration, family history of diabetes, and antidiabetic medication use, are presented in Table 1. No significant differences were observed between the treatment and placebo groups with respect to these characteristics. Dietary intake assessments revealed no significant differences in caloric consumption between groups at baseline (treatment: 2409.13 ± 743.47 kcal/day; control: 2214.71 ± 529.13 kcal/day; *p* = 0.129 and *p* = 0.278, respectively). As presented in Table 2, statistical analysis revealed no significant within‐group or between‐group changes in the total caloric intake or macronutrient distribution over the study period (*p* > 0.05). To ensure the robustness of the findings, dietary caloric intake was included as a covariate in the final adjusted LMM to adjust for its potential influence on metabolic and inflammatory outcomes. Similarly, physical activity levels remained stable across both groups, with no significant differences observed over time. Although participants were instructed to maintain their habitual physical activity throughout the study, the International Physical Activity Questionnaire (IPAQ) was administered at both time points to monitor for potential confounders. No significant differences in physical activity levels were observed between or within groups from baseline to endpoint (*p* > 0.05), confirming that physical activity did not serve as a confounding variable in this study.

**TABLE 1:** Baseline characteristics of the study patients.

Characteristics		Treatment group, *n* = 52	Placebo group, *n* = 50	*p* ^∗^
Age (years), mean ± SD		68.04 ± 3.26	67.64 ± 2.53	0.492

Sex, *n* (%)	Male	25 (48.1)	16 (32.0)	0.099
	Female	27 (51.9)	34 (68.0)	

Education, *n* (%)	Illiterate	13 (25.0)	4 (8.0)	0.080
	Primary school	23 (44.2)	22 (44.0)	
	Secondary school	9 (17.3)	23 (46.0)	
	College	7 (13.5)	1 (2.0)	

Occupation, *n* (%)	Unemployed	0 (0.0)	5 (10.0)	0.293
	Employed	8 (15.4)	0 (0.0)	
	Housewife	24 (46.2)	33 (66.0)	
	Retired	20 (38.5)	12 (24.0)	

Family history (%)	Yes	36 (69.2)	35 (70.0)	0.993
	No	16 (30.8)	15 (30.0)	

Duration of diabetes (years), mean ± SD		13.31 ± 8.20	12.84 ± 6.89	0.754

Antidiabetic medications, *n* (%)	Biguanide	20 (38.4)	18 (36.0)	0.256
	Sulfonylureas	2 (3.8)	4 (8.0)	
	Thiazolidinediones	17 (32.7)	15 (30.0)	
	Insulin injection	7 (13.5)	8 (16.0)	
	Insulin injection + oral drug	6 (11.6)	7 (14.0)	

Other medications, *n* (%)	ACE inhibitors	8 (15.4)	10 (20.0)	0.441
	Antiplatelet agents	9 (17.3)	6 (12.0)	
	β‐Blockers	12 (23.1)	10 (20.0)	
	Statin	5 (9.6)	7 (14.0)	
	ACE inhibitors + statin	5 (9.6)	4 (8.0)	

	β‐Blockers + statin	3 (5.8)	5 (10.0)	
	Antiplatelet agents + β‐blockers + statin	10 (19.2)	8 (16.0)	

Past medical history	Hypertension	40 (76.9)	40 (80.0)	0.367
	Hypothyroidism	13 (25.0)	9 (18.0)	
	Coronary Vessel Dis.	23 (44.2)	18 (36.0)	
	COPD	38 (73.1)	1 (2.0)	

Physical activity level, *n* (%)	Light	49 (94.2)	48 (96.0)	0.891
	Moderate	3 (5.8)	2 (4.0)	

Weight (kg)		76.98 ± 12.80	79.32 ± 7.36	0.259

Body mass index (kg/m^2^)		29.79 ± 4.43	31.28 ± 3.56	0.067

Fasting blood glucose (mg/dL)	≤ 100	5 (9.6)	5 (48.0)	0.800
	101–200	43 (82.7)	42 (48.0)	
	≥ 201	4 (7.7)	3 (4.0)	

^∗^Independent *t*‐test or chi‐squared and ± Fisher’s exact test.

**TABLE 2:** Comparison of the dietary intake between study groups before and after intervention.

Characteristics	Treatment group, *n* = 52	Placebo group, *n* = 50	*p* ^∗^
Energy (kcal), mean ± SD			
Before intervention	2409.13 ± 743.47	2214.71 ± 529.13	0.205
After intervention	2347.44 ± 578.51	2189.77 ± 618.45	0.189
Carbohydrate (g), mean ± SD			
Before intervention	350.78 ± 100.77	334.17 ± 54.12	0.257
After intervention	348.01 ± 77.87	328.82 ± 71.13	0.149
Protein (g), mean ± SD			
Before intervention	68.78 ± 15.15	59.13 ± 7.47	0.317
After intervention	69.13 ± 20.48	61.20 ± 17.18	0.243
Total fat (g), mean ± SD			
Before intervention	81.22 ± 31.10	71.17 ± 31.55	0.152
After intervention	75.73 ± 21.12	70.45 ± 35.12	0.083
Saturated fatty acids (g), mean ± SD			
Before intervention	19.55 ± 9.19	18.32 ± 10.01	0.489
After intervention	21.32 ± 12.46	16.43 ± 5.75	0.608
Monounsaturated fatty acids (g), mean ± SD			
Before intervention	20.01 ± 12.44	18.11 ± 11.38	0.319
After intervention	22.82 ± 18.55	20.25 ± 6.61	0.338
Polyunsaturated fatty acids (g), mean ± SD			
Before intervention	39.11 ± 19.03	37.18 ± 17.53	0.217
After intervention	32.73 ± 23.71	33.73 ± 11.11	0.108

^∗^Independent sample *t*‐test.

### 3.2. Primary and Secondary Outcomes

#### 3.2.1. Inflammatory and Oxidative Stress Markers

As presented in Table 3, at the end of the study, the treatment group demonstrated significant reductions in hs‐CRP levels, indicating a decrease in systemic inflammation (mean difference [MD]: −0.98 μg/mL; *p* = 0.034), with a moderate effect size (Cohen’s d = 0.45). This reduction in hs‐CRP is clinically meaningful, as it correlates with a lower risk of cardiovascular events and other inflammation‐related conditions.

**TABLE 3:** Comparison of serum concentrations of inflammatory and oxidative stress indices between study groups before and after intervention.

Variable		Treatment group, *n* = 52	Placebo group, *n* = 50	*P*	*q*
hs‐CRP (μg/mL), mean ± SD	Before intervention	8.64 ± 2.73	8.75 ± 1.29	**0.034**	**0.044**
	After intervention	7.66 ± 1.86	8.70 ± 2.98		
	MD (95% CI)^a^	**−0.98 (-1.92, −0.04)**	−0.05 (−1.05, +0.94)		

TAC (mmol/L), mean ± SD	Before intervention	2.97 ± 1.24	3.30 ± 1.33	**0.026**	**0.033**
	After intervention	3.58 ± 1.34	3.16 ± 1.17		
	MD (95% CI)^a^	**+0.60 (+0.21, +1.00)**	−0.13 (−0.56, +0.28)		

MDA (mmol/mL)	Before intervention	5.78 ± 1.72	5.56 ± 1.93	**0.031**	**0.038**
	After intervention	5.27 ± 1.82	5.96 ± 1.76		
	MD (95% CI)^a^	−0.50 (−1.10, +0.10)	+0.39 (−0.20, +0.99)		

*Note:* The bold values indicate statistical significance. Values for MD (95% CI) represent within‐group changes from baseline to postintervention, whereas the reported *p* values and *q*‐values refer to the adjusted between‐group comparisons derived from the adjusted models. MDA: malondialdehyde.

Abbreviations: hs‐CRP, high‐sensitivity C‐reactive protein; MD (95% CI), mean difference (95% confidence interval); TAC, total antioxidant capacity.

^a^Paired sample *t*‐test.

Additionally, a significant increase in TAC was observed (MD: +0.60 mmol/L; *p* < 0.05), reflecting enhanced antioxidant defense mechanisms, with a small‐to‐medium effect size (Cohen’s d = 0.35). After adjustment for multiple comparisons using the Benjamini–Hochberg procedure, these changes remained statistically significant in the final between‐group analysis (*q* < 0.05). This improvement in TAC suggests a potential protective effect against oxidative stress, which is relevant to overall health outcomes.

Although MDA levels decreased in the intervention group (MD: −0.50 mmol/mL; 95% CI: −1.10, +0.10), this change did not reach statistical significance in the final between‐group analysis (*p* > 0.05).

#### 3.2.2. Anthropometric Indices

As presented in Table 4, the treatment group demonstrated significant reductions in weight (MD: −1.73 kg; *p* < 0.001), with a large effect size (Cohen’s d = 0.78), and BMI (MD: −0.67 kg/m^2^; *p* < 0.001) compared to the placebo group in the final between‐group analysis, after adjustment for baseline values and energy intake. After adjustment for multiple comparisons using the Benjamini–Hochberg procedure, these changes remained statistically significant in the final between‐group analysis (*q* = 0.002). These findings indicate that the intervention was effective in reducing both weight and BMI, which are associated with improved metabolic health and a lower risk of chronic disease.

**TABLE 4:** Comparison of anthropometric and body composition indices between groups before and after intervention.

Variable		Treatment group, *n* = 52	Placebo group, *n* = 50	*P*	*q*
Weight, mean ± SD	Before intervention	76.98 ± 12.80	79.32 ± 7.36	< 0.001	0.002
	After intervention	75.25 ± 12.51	79.18 ± 7.49		
	MD (95% CI)^a^	**−1.73 (−2.32, −1.13)**	−0.14 (−0.45, +0.17)		

BMI, mean ± SD	Before intervention	29.79 ± 4.43	31.28 ± 3.56	0.009	0.004
	After intervention	29.12 ± 4.29	31.23 ± 3.66		
	Change±, MD (95% CI)^a^	**−0.67 (−0.91, −0.44)**	−0.04 (−0.17, +0.07)		

*Note:* The bold values indicate statistical significance. Values for MD (95% CI) represent within‐group changes from baseline to postintervention, whereas the reported *p* values and *q*‐values refer to the adjusted between‐group comparisons derived from the adjusted models.

Abbreviations: BMI, body mass index; MD (95% CI), mean difference (95% confidence interval).

^a^Paired sample *t*‐test.

However, no significant differences were observed in waist circumference or body fat percentage between the two groups in the final between‐group analysis (*p* > 0.05). The absence of significant change in waist circumference may suggest that, while overall weight and BMI improved, the distribution of body fat did not shift substantially within the intervention period.

#### 3.2.3. Blood Glucose Level Outcomes

As detailed in Table 5, the intervention group exhibited significant reductions in FPG (MD: −22.83 mg/dL; *p* = 0.015), with a large effect size (Cohen’s d = 0.87), indicating a substantial improvement in glycemic control. Additionally, insulin levels decreased significantly (MD: −0.94 μIU/mL; *p* = 0.008), with a moderate effect size (Cohen’s d = 0.55), suggesting enhanced insulin sensitivity in the intervention group. Furthermore, HOMA‐IR showed a significant decrease (MD: −1.31; *p* = 0.001), with a large effect size (Cohen’s d = 0.82), reinforcing the favorable impact of the intervention on metabolic health. The Benjamini–Hochberg procedure was applied to adjust for multiple comparisons; following this correction, significant improvements were maintained for FPG (*q* = 0.022), insulin (*q* = 0.013), and HOMA‐IR (*q* = 0.015) in the final between‐group analysis.

**TABLE 5:** Comparison of glucose metabolism indices between study groups before and after intervention.

Variable		Treatment group, *n* = 52	Placebo group, *n* = 50	*P*	*q*
FPG (mg/dL), mean ± SD	Before intervention	173.52 ± 37.10	169.10 ± 36.70	**0.015**	**0.022**
	After intervention	150.68 ± 28.47	166.79 ± 24.40		
	MD (95% CI)^a^	**−22.83 (−31.30, −14.36)**	−2.31 (−11.60, +6.97)		

Insulin (μIU/mL), mean ± SD	Before intervention	15.12 ± 2.74	15.51 ± 2.68	**0.008**	**0.013**
	After intervention	14.17 ± 2.71	15.38 ± 2.10		
	MD (95% CI)^a^	**−0.94 (−1.48, −0.39)**	−0.13 (−0.69, +0.43)		

HbA1C	Before intervention	7.35 ± 1.02	7.45 ± 0.87	0.313	0.418
	After intervention	7.19 ± 1.01	7.38 ± 0.91		
	MD (95% CI)^a^	−0.16 (−0.34, +0.02)	−0.06 (−0.16, +0.02)		

HOMA‐IR	Before intervention	6.66 ± 2.49	6.64 ± 2.32	**0.001**	**0.015**
	After intervention	5.34 ± 1.66	6.39 ± 1.51		
	MD (95% CI)^a^	**−1.31 (−1.75, −0.86)**	−0.24 (−0.74, +0.25)		

*Note:* The bold values indicate statistical significance. Values for MD (95% CI) represent within‐group changes from baseline to postintervention, whereas the reported *p* values and *q*‐values refer to the adjusted between‐group comparisons derived from the adjusted models. HbA1C: hemoglobin A1C, HOMA‐IR: homeostasis model assessment‐estimated insulin resistance.

Abbreviations: FPG, fasting plasma glucose; MD (95% CI), mean difference (95% confidence interval).

^a^Paired sample *t*‐test.

Although a numerical reduction in HbA1c was observed in the intervention group, this did not reach statistical significance (MD: −0.16; 95% CI: −0.34, +0.02; *p* > 0.05) and therefore cannot be interpreted as a clinically meaningful change. The absence of a significant change in HbA1c may be attributable to the relatively short duration of the intervention, as HbA1c reflects average glycemic control over a 2–3‐month period and may require a longer follow‐up to detect measurable changes. Future studies with extended intervention periods are warranted to determine whether the improvements observed in FPG, insulin, and HOMA‐IR are sustained and ultimately reflected in HbA1c levels.

#### 3.2.4. Lipid Profile

As shown in Table 6, the between‐group analysis, adjusted for baseline values, revealed a significant reduction in triglyceride levels in the intervention group compared to that of the control group (MD: −15.00 mg/dL; *p* = 0.020), with a moderate effect size (Cohen’s d = 0.54). This reduction is clinically meaningful, as lowering triglyceride levels is associated with a decreased risk of cardiovascular disease. Additionally, total cholesterol levels showed a significant between‐group difference (MD: −2.52 mg/dL; *p* = 0.028), although the effect size was small (Cohen’s d = 0.32). The Benjamini–Hochberg procedure was applied to adjust for multiple comparisons; following this correction, significant improvements were maintained in the final between‐group analysis for triglycerides (*q* = 0.029) and total cholesterol (*q* = 0.041). No significant differences were observed between groups for LDL‐C or HDL‐C levels (*p* > 0.05). These findings suggest that while the intervention effectively reduced triglyceride and total cholesterol levels, it did not significantly alter LDL‐C or HDL‐C concentrations over the study period.

**TABLE 6:** Comparison of lipid profiles between the groups before and after intervention.

Variable		Treatment group, *n* = 52	Placebo group, *n* = 50	*p*	*q*
TG (mg/dL), mean ± SD	Before intervention	142.77 ± 72.38	133.58 ± 80.48	**0.020**	**0.029**
	After intervention	127.87 ± 36.20	145.78 ± 85.44		
	MD (95% CI)^a^	−15.00 (−34.24, +4.42)	+12.19 (−0.90, +25.29)		

LDL‐C (mg/dL), mean ± SD	Before intervention	125.53 ± 26.17	135.01 ± 28.94	0.158	0.204
	After intervention	127.29 ± 31.27	140.55 ± 35.42		
	MD (95% CI)^a^	+1.75 (−9.87, +13.38)	+5.53 (−2.43, +13.50)		

HDL‐C (mg/dL), mean ± SD	Before intervention	43.18 ± 7.69	44.51 ± 15.99	0.278	0.430
	After intervention	41.57 ± 11.31	44.19 ± 9.83		
	MD (95% CI)^a^	−1.61 (−5.27, +2.04)	−0.34 (−4.06, +3.37)		

Total cholesterol, mean ± SD	Before intervention	197.02 ± 29.50	206.24 ± 32.82	**0.028**	**0.041**
	After intervention	194.49 ± 31.22	213.87 ± 40.51		
	MD (95% CI)^a^	−2.52 (−14.22, +9.18)	+7.62 (−1.91, +17.17)		

*Note:* The bold values indicate statistical significance. Values for MD (95% CI) represent within‐group changes from baseline to postintervention, whereas the reported *p* values and *q*‐values refer to the adjusted between‐group comparisons derived from the adjusted models. TG: triglycerides.

Abbreviations: HDL‐C, high‐density lipoprotein cholesterol; LDL‐C, low‐density lipoprotein cholesterol; MD (95% CI), mean difference (95% confidence interval).

^a^Paired sample *t*‐test.

#### 3.2.5. Quality of Life

As shown in Table 7, significant improvements were observed in the intervention group across several dimensions of health‐related quality of life. Specifically, physical functioning improved significantly (*p* = 0.045), with a small effect size (Cohen’s d = 0.40), indicating that participants experienced an enhanced ability to perform daily activities. Additionally, role limitations due to physical health decreased significantly (*p* = 0.041), with a small effect size (Cohen’s d = 0.39), suggesting that the intervention positively affected participants′ ability to engage in work or other essential roles.

**TABLE 7:** Comparison of the quality of life between treatment and placebo groups before and after intervention.

Variable		Treatment group, *n* = 52	Placebo group, *n* = 50	*p* ^∗∗^
Physical function, median (range)	Before intervention	87.50 (55.00)	45.00 (90.00)	**< 0.001**
	After intervention	85.00 (50.00)	50.00 (90.00)	
	*p* ^∗^	**0.045**	**0.037**	

Role disorder due to physical health, median (range)	Before intervention	62.50 (62.50)	100.00 (100.00)	**0.007**
	After intervention	62.50 (50.00)	50.00 (100.00)	
	*p* ^∗^	**0.041**	0.089	

Role disorder due to emotional health, Median (range)	Before intervention	66.66 (100.00)	100.00 (100.00)	0.155
	After intervention	100.00 (66.67)	66.67 (100.00)	
	*p* ^∗^	0.563	0.853	

Energy/fatigue, median (range)	Before intervention	50.00 (30.00)	60.00 (55.00)	0.367
	After intervention	50.00 (30.00)	60.00 (45.00)	
	*p* ^∗^	0.306	0.665	

Emotional well‐being, median (range)	Before intervention	60.00 (48.00)	72.00 (72.00)	0.435
	After intervention	60.00 (40.00)	56.00 (44.00)	
	*p* ^∗^	0.265	0.422	

Social function, median (range)	Before intervention	50.00 (50.00)	50.00 (87.50)	0.107
	After intervention	62.50 (62.50)	62.50 (75.00)	
	*p* ^∗^	0.087	0.289	

Pain, median (range)	Before intervention	32.50 (90.00)	78.75 (77.50)	**< 0.001**
	After intervention	25.00 (90.00)	55.00 (55.00)	
	*p* ^∗^	**< 0.001**	0.843	

General health, median (range)	Before intervention	50.00 (40.00)	40.00 (60.00)	**0.005**
	After intervention	50.00 (40.00)	45.00 (50.00)	
	*p* ^∗^	0.017	0.498	

*Note:* The bold values indicate the statistical significance. The score range for each subscale of QoL is 0–100. Physical health is obtained from the sum of the subscales of physical, pain, role physical, and general health. Mental health is obtained from the sum of the subscales of emotional well‐being, vitality, role emotional, and social functioning.

^∗^Mann–Whitney U.

^∗∗^Wilcoxon’s signed rank test.

Notably, participants also reported significant reductions in pain (*p* < 0.001), with a large effect size (Cohen’s d = 0.85), reflecting a substantial improvement in comfort and overall quality of life. Furthermore, general health perceptions improved significantly (*p* = 0.017), with a small‐to‐medium effect size (Cohen’s d = 0.47), indicating a positive shift in participants′ perceptions of their overall health status.

However, no significant differences were found in emotional well‐being, social functioning, vitality (fatigue), or role limitations due to emotional problems (*p* > 0.05). The lack of significant changes in these areas may suggest that while the physical aspects of health improved, the intervention did not sufficiently address emotional or social dimensions of well‐being.

#### 3.2.6. Safety and Adverse Events

The supplements were generally well tolerated among participants. To ensure the safety of high‐dose vitamin D3 supplementation in this population, serum calcium levels and renal function markers (e.g., serum creatinine) were assessed at baseline and at the end of the study. No cases of hypercalcemia or clinically significant changes in renal function were observed. Adverse effects were minor and infrequent; specifically, two individuals in the treatment group reported transient loose stools, which resolved spontaneously. No allergic reactions or serious adverse events were reported, suggesting a favorable safety profile for this cosupplementation regimen.

## 4. Discussion

This clinical trial is the first to evaluate the combined effects of flaxseed oil and high‐dose vitamin D3 supplementation in elderly patients with T2DM. Our findings indicate that 8 weeks of cosupplementation led to statistically significant reductions in hs‐CRP and improvements in oxidative stress biomarkers (TAC and MDA) compared to the placebo group. From a clinical perspective, the reduction in inflammatory markers is particularly noteworthy, as chronic inflammation is a key driver of complications in elderly patients with diabetes. It should be noted that MDA reflects lipid peroxidation specifically and does not capture the full spectrum of oxidative stress; however, the concurrent measurement of TAC in this study partially compensates for this limitation by providing a global measure of antioxidant defense capacity.

Additionally, statistically significant improvements were observed in body weight, BMI, and several metabolic markers, including FPG, HOMA‐IR, and lipid profiles (triglycerides and total cholesterol). Nevertheless, it is important to distinguish between statistical significance and clinical meaningfulness in interpreting these findings. While the reductions in total cholesterol and anthropometric outcomes were statistically significant, the magnitude of change was modest. Given the 8‐week duration of the intervention, these findings should be regarded as preliminary evidence of a favorable trend rather than a definitive clinical reversal of metabolic dysfunction.

Notably, HbA1c, LDL‐C, and HDL‐C did not change significantly; therefore, no meaningful changes in these markers can be inferred from this study. The absence of significant change in HbA1c is likely attributable to the short intervention duration, as this biomarker reflects average glycemic control over a 2–3‐month period. Longer‐term interventions are warranted to determine whether the improvements observed in FPG and HOMA‐IR are ultimately reflected in HbA1c levels.

These results are consistent with a growing body of literature documenting the beneficial effects of combined vitamin D and omega‐3 fatty acid supplementation on metabolic and inflammatory markers. Previous studies involving patients with T2DM and CHD have reported similar benefits, indicating that such cosupplementation may contribute to improved cardiometabolic health [14]. Furthermore, interventions in diverse populations, including those with colorectal cancer and gestational diabetes, have demonstrated reductions in inflammatory markers and improvements in oxidative stress profiles [18, 37].

The observed reductions in hs‐CRP and improvements in oxidative stress biomarkers underscore the anti‐inflammatory potential of this cosupplementation strategy. Chronic inflammation is recognized as a significant contributor to the pathogenesis of T2DM, and the ability of flaxseed oil and vitamin D3 to attenuate inflammatory responses suggests a potential supportive role in managing inflammation in elderly patients, who are particularly susceptible to heightened inflammatory activity and oxidative stress.

Mechanistically, the synergistic effects of vitamin D and omega‐3 PUFAs may be attributed to their roles in modulating inflammatory markers. The anti‐inflammatory, antiangiogenic, and proapoptotic properties of these compounds may act in concert to enhance metabolic health [38]. Large‐scale trials have suggested that supplementation with vitamin D and omega‐3 fatty acids can reduce the risk of autoimmune diseases and cardiovascular events, particularly in populations with a low dietary intake of fish and omega‐3 fatty acids [39, 40]. Notably, studies conducted in Iran have demonstrated significant improvements in glycemic outcomes and lipid profiles among women with prediabetes and other metabolic disorders when higher doses of vitamin D and omega‐3 fatty acids are used [14].

The variability in the efficacy of combined supplementation may depend on baseline characteristics such as BMI and dietary habits, which could explain inconsistencies observed in previous studies [40]. For instance, while flaxseed oil is a rich source of ALA, its effectiveness in raising the omega‐3 index—which includes eicosapentaenoic acid (EPA) and docosahexaenoic acid (DHA)—is limited compared to marine‐derived omega‐3 sources, as the endogenous conversion of ALA to EPA and DHA in humans is well documented to be low and highly variable [41, 42]. This underscores the need for a nuanced understanding of dietary sources and their respective effects on health outcomes [43].

Our findings align with previous research indicating that flaxseed oil and vitamin D3 supplementation can significantly influence metabolic parameters; however, they also highlight the mixed results reported in the literature regarding glycemic outcomes. While some studies have demonstrated beneficial effects on FBG and insulin sensitivity [14], others have reported negligible changes with high‐dose flaxseed oil alone [15]. These discrepancies may stem from variations in study design, participant demographics, and intervention duration.

Even mild hyperglycemia can trigger inflammatory processes that exacerbate insulin resistance, which frequently coexists with metabolic syndrome—a condition defined by the presence of at least three of five diagnostic criteria, including abdominal obesity, elevated triglycerides, reduced HDL‐C, elevated blood pressure, and elevated FBG [44]. Our findings reinforce the notion that targeted nutritional interventions, such as supplementation with vitamin D and omega‐3 fatty acids, may modulate these underlying inflammatory pathways under specific study conditions [14].

Oxidative stress also plays a crucial role in the development of T2DM. Chronic hyperglycemia promotes the accumulation of reactive oxygen and nitrogen species, contributing to insulin resistance and beta‐cell dysfunction [45, 46]. Nutritional interventions targeting oxidative stress, such as supplementation with vitamin D and omega‐3 fatty acids, may offer therapeutic benefits [47].

Vitamin D deficiency is prevalent in elderly individuals and is associated with an increased risk of T2DM [48]. Supplementation may improve glucose homeostasis, reduce oxidative stress, and minimize DNA damage in patients with diabetes [49]. Long‐term vitamin D supplementation has been shown to reduce fasting insulin levels and inflammatory markers; however, its effects on cardiovascular outcomes and renal function in older populations with diabetes remain inconclusive [50, 51].

The evaluation of quality of life in this trial adds another dimension of significance to the findings. The improvements observed in physical health domains—such as reduced pain scores, enhanced general health perceptions, and improved physical functioning—suggest that the metabolic and anti‐inflammatory benefits of the supplementation may translate into better overall well‐being for elderly patients with T2DM. Although no significant differences were observed in mental health–related domains, the nonsignificant trends toward improvement warrant further investigation, as mental health is influenced by a complex interplay of factors beyond nutritional interventions.

The improvement in physical aspects of quality of life observed in this study is consistent with findings from previous research examining the effects of vitamin D and omega‐3 fatty acid supplementation on quality of life in patients with chronic diseases, including T2DM. For instance, a study by Jamilian et al. [52] on women with polycystic ovary syndrome (PCOS) demonstrated that cosupplementation with omega‐3 fatty acids and vitamin D significantly improved general health perceptions and reduced bodily pain scores compared to placebo, consistent with the physical health improvements observed in this trial. In contrast, several studies have reported varied or no significant effects on mental health–related quality of life domains, in line with the present findings. For example, Mahmoodi et al. [53] reported no significant changes in emotional well‐being or social functioning following vitamin D supplementation in patients with T2DM, despite improvements in metabolic indicators. These findings suggest that mental health domains may be less responsive to short‐term nutritional interventions and more influenced by psychosocial, behavioral, and environmental factors.

In summary, the mechanistic interpretation of the study findings highlights a synergistic effect of flaxseed oil and vitamin D3 in modulating inflammatory processes, enhancing antioxidant defenses, improving glycemic outcomes, and favorably influencing lipid profiles, within the limits of the current trial’s duration and design. Further long‐term studies are warranted to confirm whether these physiological changes translate into definitive clinical practice recommendations for elderly patients with T2DM.

This study has several strengths. It is the first randomized, placebo‐controlled trial to assess the combined effects of flaxseed oil and high‐dose vitamin D3 in elderly patients with T2DM, addressing a significant gap in the literature. The study design minimized potential biases, enhancing the internal validity of the findings. A comprehensive range of metabolic, inflammatory, and oxidative stress markers was measured, providing a thorough assessment of the intervention’s effects. The dose of vitamin D3 used was sufficient to correct potential deficiencies, particularly in an elderly population at a higher risk of insufficiency. The favorable safety profile observed supports the feasibility of this cosupplementation strategy in clinical practice.

However, several limitations should be considered when interpreting the findings. First, the relatively short intervention duration (8 weeks) may have limited the ability to detect changes in long‐term outcomes such as HbA1c and lipid fractions, which typically require longer follow‐up periods to show meaningful change. Second, although systemic inflammatory and oxidative stress markers were measured, the study did not include mechanistic or tissue‐specific markers that could elucidate the underlying biological pathways through which the intervention exerted its effects. Third, while dietary intake was assessed at both baseline and end of intervention using a validated food frequency questionnaire, macronutrient data were collected as absolute amounts (grams per day) rather than as percentage contributions to total energy intake, which provides a less precise characterization of the overall dietary quality. Fourth, physical activity was monitored via self‐report, and participants were instructed to maintain their habitual activity levels throughout the study; however, formal records of activity type, frequency, and duration were not collected, and objective monitoring tools such as accelerometry were not employed. Consequently, a training effect cannot be entirely ruled out as a potential source of residual confounding. Fifth, although baseline serum 25‐hydroxyvitamin D was measured for eligibility screening, postintervention serum 25‐hydroxyvitamin D concentrations were not assessed, which limited biochemical confirmation of vitamin D response and compliance. Sixth, the study sample comprised older adults with Type 2 diabetes from a specific clinical setting, which may limit the generalizability of the findings to other populations or ethnic groups. Seventh, BMI was used as the sole anthropometric measure. Central adiposity indices such as waist circumference or waist‐to‐height ratio are more closely associated with visceral fat and metabolic outcomes, including glycemic control and inflammatory markers; future research should incorporate these measures. A further limitation concerns the use of MDA as a marker of oxidative stress. MDA is a byproduct of lipid peroxidation and reflects only one dimension of oxidative damage, without capturing protein oxidation, DNA oxidation, or other aspects of the broader oxidative stress response. More comprehensive markers, such as 8‐isoprostane, protein carbonyl content, or 8‐hydroxy‐2′‐deoxyguanosine (8‐OHdG), would provide a more complete assessment of total oxidative burden. However, MDA was selected alongside TAC because of its widespread use in clinical nutrition research and its established sensitivity to dietary fatty acid interventions. Future studies should incorporate a broader panel of oxidative stress biomarkers to more fully characterize the antioxidative effects of this cosupplementation. A further limitation is the absence of postintervention serum 25‐hydroxyvitamin D measurements. Although baseline vitamin D levels were assessed as part of the eligibility criteria, endpoint measurements were not obtained, which precludes biochemical confirmation that circulating vitamin D levels increased in response to supplementation. Without this, it is not possible to verify that the observed effects were attributable to the correction of vitamin D status. Similarly, plasma ALA and EPA concentrations were not measured at any time point, making it impossible to confirm adequate absorption or conversion of flaxseed oil–derived ALA. Compliance was assessed through returned pill and capsule counts, which is acknowledged as an unreliable method that cannot substitute for objective biochemical verification. These gaps represent meaningful constraints on the interpretability of the findings and should be addressed in future studies through measurement of postintervention 25‐hydroxyvitamin D levels and plasma fatty acid profiles. Finally, the long‐term sustainability of the observed benefits following discontinuation of supplementation remains unknown and warrants investigation in future research.

## 5. Conclusion

This is the first study to evaluate the combined effects of flaxseed oil and high‐dose vitamin D3 supplementation in elderly patients with T2DM. The findings suggest that this cosupplementation may serve as a promising adjunct to modify several key health indicators in this population. Specifically, significant improvements were observed in selected markers of inflammation and oxidative stress, as evidenced by favorable changes in hs‐CRP, TAC, and MDA levels. The intervention was also associated with beneficial metabolic trends, including reductions in body weight and BMI, as well as improvements in FBG, insulin resistance, and lipid profiles. Furthermore, participants reported improved scores in physical domains of quality of life, particularly with respect to pain and physical functioning. While these results are encouraging, they should be interpreted with caution given the study’s limited duration and sample size. Long‐term, large‐scale trials are warranted to determine the clinical significance and lasting effects of this intervention on HbA1c, lipid profiles, and cardiovascular outcomes in this population.

## Author Contributions

N.D. contributed to the design of the study, implementation of the intervention, clinical follow‐up of participants, data collection, and drafting of the final manuscript. M.A.O. was responsible for screening potential participants, implementing the intervention, collecting data, writing the report, and extracting and analyzing results. N.D. was responsible for designing the trial protocol, writing the protocol, extracting and analyzing data, interpreting results, and drafting the initial and final manuscript. A.F.K. contributed to the preparation, revision, and critical review of the manuscript on behalf of the original research group.

## Funding

Funding was received from the Deputy of Research, Faculty of Medicine, Tabriz University of Medical Sciences, Tabriz, Iran (No. 71371).

## Disclosure

All authors have read and approved the final manuscript. The study funders had no role in the study design, collection, analysis, and interpretation of data, the report’s writing, or the decision to submit the article for publication.

## Conflicts of Interest

The authors declare no conflicts of interest.

## Data Availability

The data that support the findings of this study are available on request from the corresponding author. The data are not publicly available due to privacy or ethical restrictions.
